# Lipid Mediators in Acne

**DOI:** 10.1155/2010/858176

**Published:** 2010-08-25

**Authors:** Monica Ottaviani, Emanuela Camera, Mauro Picardo

**Affiliations:** Laboratory of Cutaneous Physiopathology, San Gallicano Dermatological Institute IRCCS, Via Elio Chianesi 53, 00144 Rome, Italy

## Abstract

Multiple factors are involved in acne pathogenesis, and sebum secretion is one of the main ones. The role sebum plays in acne development has not been completely elucidated yet; however, increasing amounts of data seem to confirm the presence of alterations in sebum from acne patients. Altered ratio between saturated and unsaturated fatty acids has been indicated as an important feature to be considered in addition to the altered amount of specific fatty acids such as linoleic acid. Furthermore, particular attention has been focused on squalene peroxide that seems to be able to induce an inflammatory response beyond cytotoxicity and comedones formation. Moreover, recent data suggest that lipid mediators are able to interfere with sebocytes differentiation and sebogenesis through the activation of pathways related to peroxisome proliferators-activated receptors. Understanding the factors and mechanisms that regulate sebum production is needed in order to identify novel therapeutic strategies for acne treatment.

## 1. Introduction

Acne is a skin disorder with multifactorial pathogenesis. The mechanisms underlying its onset and subsequent development are not yet completely clarified. Among the factors implicated in acne occurrence, sebum secretion can be considered as the major one. Increased sebum secretion is a characteristic of acne patients even if seborrhea *per se *is not a sufficient condition for the development of the pathology. Even if it is generally accepted that a correlation exists between facial sebum outflow and acne severity [1–4], it has been suggested more recently that seborrhoea does not strictly correlate with the development of the lesions but it affects their inflammatory changes [5]. Along with the increased sebum secretion, several qualitative modifications have been described in acne patients, underlying the pivotal role played by lipid mediators derived from sebum alterations in acne pathogenesis. 

## 2. Sebum

**Figure 1:**
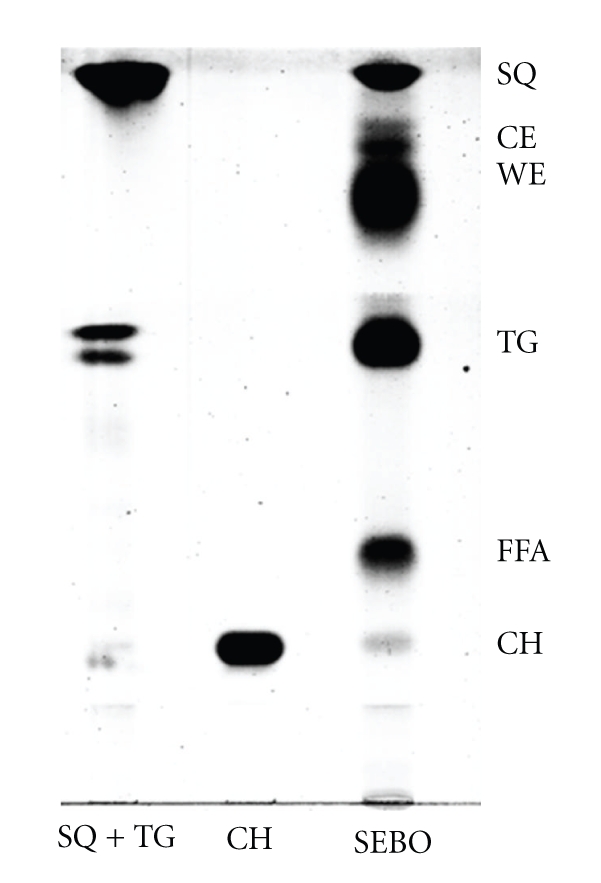
*Thin Layer Chromatography of forehead sebum.* Triglycerides (TG); Free fatty acids (FFA); Wax esters (WE); Squalene (SQ); Cholesterol esters (CE); Cholesterol (CH).

Human sebum is the holocrine secretion formed by the complete disintegration of glandular cells into the follicular duct of the pilosebaceous unit. Sebocytes are specialized cells that synthesise lipids and accumulate them in cytoplasmic lipid droplets. During the final stages of differentiation, when they become fully mature, sebocytes undergo disintegration and release their content, an oily and waxy material, into the follicle [6–9]. Human sebum contains triglycerides, wax esters, squalene, cholesterol esters, cholesterol, and free fatty acids. Triglycerides and fatty acids, taken together, account for the predominant proportion (57,5%), followed by wax esters (26%) and squalene (12%). The least abundant lipid in sebum is cholesterol, which with its esters, accounts for the remaining 4,5% of total lipids (Table 1 and Figure 1) [10]. Due to the complex composition, different hypotheses have been formulated to explain the ultimate function of sebum. Sebaceous lipids contribute to the integrity of the skin barrier supplying the stratum corneum with the hydration [11, 12], providing photo-protection, particularly against UVB [13] and delivering lipophilic antioxidants to the skin surface [14]. Moreover, sebaceous gland lipids have been demonstrated to have pro- and anti-inflammatory properties [15, 16], and some specific lipids, such as oleic and palmitoleic acid, have been hypothesized to exert an antibacterial effect [17]. However, the exact role of human sebum as well as the metabolic pathways regulating its composition and secretion has not been clarified so far. Altogether, the roles played by sebum modifications and alterations in the onset and development of acne remain to be elucidated. 

## 3. Sebum Alterations and Acne

Quantitative and qualitative variations have been detected in sebum from acne patients. The production of sebum is an indispensable condition for the occurrence of acne, however, sebum hypersecretion *per se* is not sufficient to trigger the disease. Experimental and clinical data seem to address some compositional modification of sebum as causative factors of clinical signs of acne. 

### 3.1. Fatty Acids: Linoleic Acid

In the early studies concerning acne, decreased amounts of linoleic acid in the skin surface lipids were observed in affected patients. In particular, levels of this essential fatty acid were found significantly lower in wax esters making it reasonable to assume that linoleic acid is directly involved in the sebaceous lipid synthesis [18]. Wax esters and squalene, in fact, are sebum-specific lipids. The uptake of circulating lipids and the *β*-oxidation activity are important steps for the production of the sebaceous ones. Presumably, fatty acids are selectively utilized by the sebaceous gland, and linoleic acid seems to be the only one subjected to *β*-oxidation. It is preferentially transformed into two carbon unit precursors leading to the generation of acetyl-CoA that are incorporated into different metabolic route such as the biosynthetic pathway leading to squalene and wax esters synthesis. In experimental models, linoleic acid *β*-oxidation seems to correlate with specific sebaceous lipid production (wax esters) and thus with sebocytes functions and differentiation [19]. 

A diminished amount of linoleic acid in the sebum has been suggested to affect the composition of sphingolipids in the follicle. Acne patients show a lower percentage of acyl-ceramides containing linoleic acid. Depletion of linoleic acid in sphingolipids has been hypothesized to be involved in the follicular hyperkeratosis, which is a crucial event involved in the comedones formation [20]. Moreover, low levels of linoleic acid also lead to impairment of the epidermal barrier function and predispose to the increased permeability of comedonal wall to inflammatory substances [21].

### 3.2. Fatty Acids: Saturated and Unsaturated Pattern

**Figure 2:**
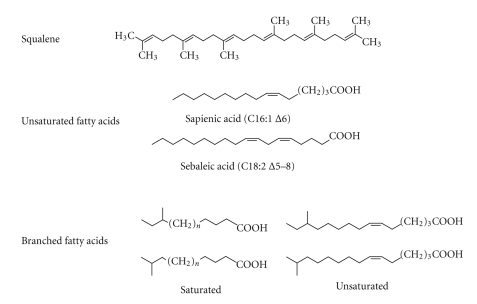
*Characteristic components of sebaceous secretion.* Squalene is a lipid exclusive of human sebaceous secretion. Among fatty acids, there are some with a peculiar pattern of unsaturation (sapienic and sebaleic acid) and some with a particular chemical structure (branched fatty acids).

Recent studies have reported a significantly different ratio between saturated and unsaturated fatty acids in acne. In particular, the C16 : 0/C16 : 1 ratio in the skin surface triglycerides and wax esters is higher in acne [22, 23]. Increased sebum outflow and severity of clinical manifestations were associated with an alteration in the proportion of monounsaturated fatty acids suggesting that desaturation of fatty acids may play a major role in sebogenesis and acne onset. The desaturase that yields unsaturated fatty acids of the type found in sebum inserts a double bond at the position Δ6 of the carbon backbone. This enzyme, known either as Δ6 desaturase or fatty acid desaturase-2 (FADS-2), is detectable mainly in differentiated sebocytes, which occupy the suprabasal layers of the sebaceous gland and have reached a full lipid synthetic capacity. Δ6 desaturase can be considered as a functional marker of activity and differentiation of sebocytes [24], and leads to the formation of fatty acids with a peculiar pattern of unsaturation. In particular, Δ6 desaturase acts on palmitic acid (C16 : 0) to produce sapienic acid (C16 : 1Δ6), the most abundant fatty acid in human sebum. This unusual fatty acid with a desaturation at the Δ6 position instead of the Δ9 position has not been identified in any other human tissue nor in sebum secretion of other animals. A fatty acid unique to sebum, such as sebaleic acid (C18 : 2Δ5,8), presents a sebaceous pattern of unsaturations consequent to the elongation by two carbons of the precursor sapienic acid and the additional insertion of a double bond (Figure 2) [24]. The ratio between Δ6 and Δ9 unsaturated fatty acids has been proposed as an index of maturation of sebaceous cells and of metabolic process associated to it [25]. Incorrect activity of specific desaturase enzymes and/or excessive sebum secretion can result in an alteration of the relative proportion of the different fatty acids, and consequently in the within-class distribution of lipids constituting sebum, leading to compositional changes that can drive towards acne development. 

### 3.3. Squalene and Squalene Peroxide

The modifications of lipid composition illustrated so far in the acne sebum are the result of an incorrect lipid synthesis in the sebaceous gland. Post synthetic compositional changes can occur due to different factors, which remain to be clarified. Among the lipids proposed as having a role in the development of comedones, particular attention has been focused on lipid peroxidation products. A recent study has demonstrated that the accumulation of lipid peroxides may be responsible for the inflammatory changes in comedones. In particular, it has been observed that the degree of lipoperoxidation as well as interleukin-1-alpha and NF-kappaB content are higher in inflammatory lesions than that in noninflammatory ones [26]. These data further support previous findings suggesting an association between sebum oxidation and acne progression, strengthening the role of lipid peroxidation in acne pathogenesis. Squalene peroxidation products continue to receive particular attention considering that squalene, together with wax esters, represents characteristic products of sebaceous secretion. In fact, squalene and wax esters have a particularly high concentration in sebum and are not found among the epidermal surface lipids. Biosynthesis of wax esters is important for the sebaceous gland survival. DGAT are key enzymes involved in sebogenesis. The DGAT1 isoform catalyzes the sysnthesis of wax esters, unlike DGAT2 that leads to triacylglycerols [27]. Squalene is a linear intermediate preceding cholesterol in its biosynthetic pathway. In the sebaceous gland conversion of squalene to lanosterol and then to cholesterol is negligible.The reason why squalene is accumulated in sebocytes still awaits further investigations. There are different possibilities that might explain the high squalene content in sebum. On one side, in sebocytes there might be an increased expression and/or activity of the enzyme that produces squalene, namely squalene synthase; on the other one the enzymes involved in the transformation of squalene into cholesterol, which include squalene-2,3-epoxidase and oxidosqualene cyclase, might be repressed. Considering that sebaceous gland possesses a peculiar environment characterized by an anaerobic condition, and that squalene-2,3-epoxidase needs oxygen to proceed in its reaction, it is reasonable to speculate that squalene accumulation in sebocytes is due to the peculiar environment of the sebaceous gland [8]. For all above mentioned, squalene may be considered as a marker of sebocytes differentiation and therefore of sebogenesis [15]. Following UV exposure, squalene undergoes massive photodegradation due to its highly unsaturated chemical structure. Irradiation of human skin leads to a squalene decomposition of about 60% similar to that observed *in vitro* on the purified compound [28]. Oxidative challenge generates, in human skin surface lipids, squalene monohydroperoxide as the main product [29]. Several optional isomers of squalene monohydroperoxide have been identified. Additional peroxidation of squalene monohydroperoxide can also occur. Altogether, squalene peroxidation byproducts exert harmful effects *in vivo* and in skin cell cultures. In particular, keratinocytes cytotoxicity [28], histological changes, and immunosuppressive effects have been observed [30]. Comedogenicity of squalene peroxides has been demonstrated in animal experiments in which comedones have been induced by exposing rabbit ears to irradiated squalene. The degree of squalene peroxidation was found to correlate positively with the size of the comedones elicited. In addition, the treatment of ear skin with squalene peroxidation by-products caused marked hyperplasia and hyperkeratosis of the epithelium in follicular infundibulum, and increased the proliferation of the sebaceous glands [31]. These effects were specifically caused by squalene monohydroperoxide whereas the all saturated squalene form (squalane), squalene itself and synthetic peroxides with different backbone structures, exerted a negligible comedogenic effect and did not lead to skin roughness and wrinkling [32, 33]. *In vitro* experiments have shown that squalene peroxides, beyond induction of keratinocytes proliferation, led also to the upregulation and release of inflammatory mediators, such as interleukine-6. Overall, these effects clearly suggest a proinflammatory activity of squalene oxidation products [34]. The onset of inflammatory reactions appears to be an early event in the development of acne lesions [35, 36]. The oxidative challenge supplied with peroxidated squalene can be aggravated by its potential glutathione-depleting activity which results in an increased cytotoxicity and comedogenicity [37]. To limit the harmful effects of peroxidated squalene, the skin is equipped with endogenous defense system. Vitamin E is a lipophilic antioxidant supplied to the skin surface through the sebum outflow. In skin areas with elevated sebaceous glands density a continuous secretion of vitamin E is observed, which is in tight correlation with the levels of cosecreted squalene. This positive correlation highlights a physiological antioxidant strategy put in place to counteract the generation of squalene oxidation products [14]. The crucial role played by squalene peroxidation in acne development is strengthened by the observation that either skin surface and comedone lipids collected from acne patients are enriched in polar lipids mainly derived from squalene oxidation [38, 39]. Moreover, recent data have further confirmed these findings. Significant differences have been detected in the levels of squalene and vitamin E in sebum from acne patients and healthy subjects. In particular, a higher amount of squalene peroxide and a decreased level of vitamin E have been detected in acne sebum [40]. This finding further strengthens a role of lipid peroxidation by-products, particularly squalene perocides, in the onset and development of acne [41, 42] (Table 2).

## 4. Sebum, Oxidative Stress, PPARs, and Inflammation

Modifications of the sebum composition, due to lipoperoxidation and anomalous distribution of fatty acids, impact keratinocytes proliferation and differentiation. Importantly, lipid peroxidation products are also capable of inducing production of pro-inflammatory cytokines and activation of peroxisome proliferators-activated receptors (PPARs). PPARs are nuclear transcription factors involved in the control of lipid metabolism as well as in the control of inflammation. Modulation of inflammatory pathways results from the antagonism of the NF-*κ*B activation and the promotion of the pro-inflammatory eicosanoids catabolism [43]. Among the different isoforms, PPAR*α* and PPAR*γ* are considered the main ones involved in the sebocytes biology. In particular, PPAR*α* seems to be related to *β*-oxidation of fatty acids and lipid catabolism, whereas PPAR*γ* activation has been linked to lipidogenesis [44]. Eicosanoid metabolites originated from the arachidonic acid cascade, namely LTB_4_ and 15-HETE, have been shown to be ligands of PPAR*α* and PPAR*γ*, respectively [45, 46]. Interestingly, the enzymes involved in their formation, including 5-lipoxygenase (5-LOX), have been found to be expressed at higher extent in acne-involved skin in comparison to the skin of healthy subjects. In addition, in acne-affected skin, enhanced expression of IL-6 and IL-8 has been also found [47]. LOX products have been implicated in inflammatory skin diseases characterized by keratinocytes hyperproliferation [48, 49]. Activation of 5-LOX results, among other effects, in induced IL-6 and IL-8 expression in human sebocytes. Systemic treatment of acne patients with a 5-LOX inhibitor reduces the inflammatory lesions count and the synthesis of sebum lipids, in particular, of those with pro-inflammatory potential [41, 50]. 5-LOX inhibitors may also downregulate the inflammatory activity of lymphocytes and macrophages resulting in cumulative beneficial effects [51].

Prostaglandins are other pro-inflammatory mediators thought to be involved in acne lesion development [52]. Mouse with increased cyclooxygenase-2 (COX-2) expression and prostaglandins E_2_ (PGE_2_) levels showed sebaceous gland hyperplasia and enhanced sebum production [53] suggesting an important role for COX-2 signaling pathway in sebocytes biology. In *in vitro* models, it has been demonstrated that expression and activation of COX-2 is PPAR*γ* mediated. General oxidative stressors, including lipid oxidizing agents, have been shown to activate PPAR*γ* and to induce lipogenesis in sebocytes [52–55]. All these findings allow to hypothesize that sebocytes proliferation and/or lipogenesis as well as inflammatory reactions may be regulated by PPAR*γ*-mediated pathways. Clinical data, demonstrating upregulated expression of both COX-2 and PPAR*γ* in acne involved skin, support this hypothesis [47] and add new insights on acne pathogenesis. Taken together, all these findings suggest a comprehensive link between inflammation and sebogenesis supporting the definition of acne as an inflammatory disease in which lipid mediators play a central role. 

## 5. Conclusion

Sebum production is considered one of the principal factors involved in acne development. Great efforts have been made and are currently devoted to studying the factors that regulate sebum composition and secretion. In particular, the pathways leading to the formation of lipids typically sebaceous, such as branched fatty acids and fatty acids with uncommon unsaturation patterns, remain to be elucidated. Modifications in the amount, type and, arrangement of fatty acids constituting sebum lipids have been observed in acne patients. By-products of lipid peroxidation, in particular squalene peroxide, have been recognized to play a crucial role in the development of inflammatory reactions as well as in cytotoxicity and comedogenesis. A number of lipid mediators have been demonstrated to be PPAR*γ* ligands affecting sebocytes biology, in particular their lipid metabolism and synthesis. Moreover, PPAR*γ* activation seems to be necessary for COX-2 signaling pathway induction. Clinical data indicating increased expression of COX-2 and PGE_2_ in acne involved skin associated with enhanced release of pro-inflammatory cytokines and a higher degree of lipoperoxidation further support the interplay between lipoinflammation and lipid signaling. However, the lack of definitive data underlines the importance of acquiring detailed information of lipid molecules specifically involved in acne pathogenesis. Improvement of the knowledge on the function of sebum, on the mechanism that regulate its production, and on the role of alteration in sebum organization represents a fundamental step for the identification of new targets for innovative therapeutic strategies aiming to correct the sebum deregulation in acne. 

## Figures and Tables

**Table 1:** *Components of skin surface lipids*. Triglycerides (TG); Free fatty acids (FFA); Wax esters (WE); Squalene (SQ); Cholesterol Esters (CE); Cholesterol (CH).

	Sebum (%)	Epidermal lipids (%)
TG	30–50	30–35
FFA	15–30	8–16
WE	26–30	—
SQ	12–20	—
CE	3.0–6.0	15–20
CH	1.5–2.5	20–25

**Table 2:** *Harmful effects of squalene peroxidation*. The adverse effects of squalene peroxidation products and in particular of squalene monohydroperoxide have been assessed both in vitro and in vivo. Moreover, sebum from acne patients seems to have high level of squalene peroxide.

Squalene peroxidation	
Harmful effects	Acne sebum

Comedogenicity	Increased amount of squalene peroxidation products accompanied by decreased level of vitamin E
Sebaceous gland	
hyperplasia	
Hyperkeratosis	
Release of inflammatory	
mediators from	
keratinocytes	

## Abbreviations

Sq: Squalene
C16 : 0: Palmitic acid
C16 : 1 Δ6: Sapienic acid
C18 : 2 Δ5,8: Sebaleic acid.
